# Cancer‐Specific health equity metrics in the United States of America: A scoping review

**DOI:** 10.1002/cam4.5881

**Published:** 2023-04-05

**Authors:** Angela Starkweather, Bevin Cohen, Tamryn F. Gray, Lauri Linder, Noah Zanville

**Affiliations:** ^1^ University of Florida College of Nursing Gainesville Florida USA; ^2^ Mount Sinai Health System New York New York USA; ^3^ Dana‐Farber Cancer Institute, Harvard Medical School Boston Massachusetts USA; ^4^ University of Utah College of Nursing, Primary Children's Hospital, Huntsman Cancer Institute Salt Lake City Utah USA; ^5^ Clinical Services Group HCA Healthcare Nashville Tennessee USA

**Keywords:** cancer outcomes, health equity, quality indicators

## Abstract

Health disparities in cancer care persist, and in some cases are growing, despite decades of research aimed at achieving equal outcomes for all Americans. There is growing consensus that reducing disparities will require a shift from aiming to provide care that is equal, to aiming to provide care that is equitable. The current landscape of metrics and interventions that move beyond equality (i.e., care provided equally to all patients) and towards equity (i.e., care provided variably and justly such that patients achieve equal outcomes) have not been characterized. Thus, the aim of this scoping literature review was to identify cancer‐specific health equity metrics and interventions, and to explore current gaps in the field. Following PRISMA guidelines, PubMed, CINAHL, PsycInfo, and Scopus were searched for studies published in English between 2012 and 2022 that implemented a metric to identify or an intervention to address cancer care inequities in the United States. The search returned 36,724 unique articles, of which 40 articles (1%) included an intervention to advance health equity. Metrics included timeliness of screening and treatment, receipt of goal‐concordant care, and survival. The vast majority of articles were cross‐sectional or cohort studies that described health disparities using one or more outcome metrics. Gaps identified included research on receipt of guideline‐concordant care, interventions addressing multiple levels of structural and social determinants of health, inclusion of children and families, and patient‐reported outcomes or other sources of data that could help inform interventions to advance equity.

## 1 INTRODUCTION

Systematic inequalities in cancer care have been noted since the early 1970s following the passage of the National Cancer Act of 1971 and the creation of the National Cancer Institute's (NCI's) Surveillance, Epidemiology, and End Results (SEER) program. Over the subsequent five decades, data from SEER have revealed disparities in screening, incidence, treatment, and outcomes for a range of communities and cancer types. Health disparity is defined as a preventable difference in the burden of disease, outcomes of treatment, and opportunity to achieve optimal health that socially disadvantaged populations experience compared to the population as a whole.^1^ Health disparities are driven by social disadvantage or social inequity, which is “the unfavorable social, economic, or political conditions that some groups of people systematically experience based on their relative position in social hierarchies”.^2^ The broad constellation of social, economic, political, and environmental factors that influence an individual's health are referred to as social determinants of health, and these directly affect health equity, or the “attainment of the highest level of health for all people”.^1^ There is growing consensus that reducing disparities will require a shift from aiming to provide care that is equal (i.e., care provided equally to all patients), to aiming to provide care that is equitable (i.e., care provided variably and justly such that patients achieve equal outcomes). However, the current landscape of metrics and interventions that move beyond equality and towards equity have not been characterized systematically.

At a time when treatment options and survivorship rates continue to improve for most major types of cancer, vast inequities in cancer incidence and receipt of guideline‐concordant treatment persist for racial and ethnic minority groups.^3^ Additionally, evidence shows that children, adolescents and young adults, the uninsured, immigrants, refugees, asylum seekers, individuals with disabilities, sexual and gender minorities, and people residing in low‐access areas continue to face hurdles in cancer screening and treatment.^4^, ^5^, ^6^

Fundamentally, disparities in cancer control, treatment, and outcomes are rooted in the unequal distribution of health determinants, that is, social‐structural factors that include institutional and living environments and include health literacy, financial status, and perception of cancer/cancer prevention.^7^, ^8^ Efforts to conceptualize the factors affecting cancer outcomes and equity in cancer care led to the development of the National Cancer Institute's research agenda that emphasized multilevel interventions for addressing the individual patient and at least two levels of contextual influence.^9^ Moreover, as part of the American Cancer Society's Cancer Control Blueprint series, Alcaraz et al.^7^ highlighted the identification of (1) cancer disparities, (2) populations with whom targeted interventions should be co‐developed, implemented and evaluated, and (3) metrics for evaluating cancer‐related health equity interventions as critical steps needed to advance the field.

A brief timeline summarizing national efforts towards cancer‐related health equity is provided as Data S1. In response to these initiatives, some successful interventions for improving health equity have emerged.^10^, ^11^, ^12^ However, the current state of such interventions has not been characterized systematically, and validated metrics for assessing the influence of cancer‐related health equity interventions remain elusive. Therefore, the purpose of this scoping review was to identify cancer‐specific health equity interventions and metrics that have been deployed in practice, to examine how these indicators have been implemented and evaluated across the cancer care continuum, and to identify current research gaps in the field.

## 2 METHODS

We followed the preferred reporting items for systematic reviews and meta‐analyses (PRISMA) statement^13^ extension for scoping reviews.^14^ The literature search was conducted from January 4, 2022, to May 17, 2022, with consultation from a professional librarian. The time frame of the literature search was prompted by the authors' review of widening disparities in cancer care caused by the COVID‐19 pandemic and compilation of research priorities.^15^ Publications from peer‐reviewed journals were searched using the databases Pubmed/Medline, PsychInfo, CINAHL, and Scopus using the following Boolean/phrase or string:

(*“Neoplasms/diagnostic imaging”[Mesh] OR “Neoplasms/economics”[Mesh] OR “Neoplasms/education”[Mesh] OR “Neoplasms/epidemiology”[Mesh] OR “Neoplasms/genetics”[Mesh] OR “Neoplasms/pathology”[Mesh] OR “Neoplasms/prevention and control”[Mesh] OR “Neoplasms/psychology”[Mesh]*) *AND health disparities OR health equity* or (*oncology OR cancer AND interventions* AND *health equity*) without filters published between 2012 to 2022.

Articles were eligible for inclusion if they were (1) full‐text, (2) English language, (3) prospective or retrospective research studies of any design, (4) implemented and/or evaluated one or more interventions designed to address inequities in cancer care using a specific metric, (5) conducted in the United States, and (6) published between 2012 and 2022. Exclusion criteria were as follows: (1) case studies, review articles, and studies focused only on identifying an inequity or disparity in cancer care. While educational programs to either increase knowledge, beliefs, and attitudes about cancer screening^16^, ^17^ or increase participation in clinical research trials^18^ have been well‐studied, we were specifically interested in methods or interventions that measured an impact on one or more cancer‐related health equity metrics.

Two authors independently screened each abstract, and discrepancies were resolved by a third author. Articles that met criteria for inclusion following full‐text review were abstracted to describe the study purpose and design, population, metric(s) and main findings.

## 3 RESULTS

**FIGURE 1:**
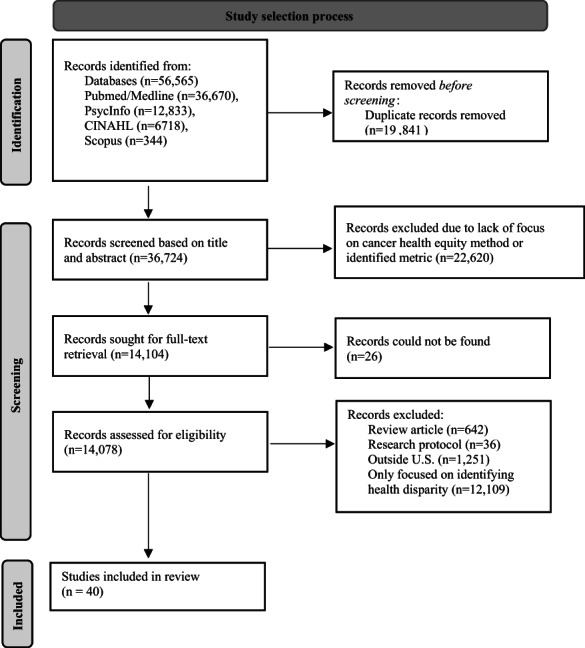
Preferred reporting items for systematic reviews and meta‐analyses flow diagram of review screening process.

A total of 14,078 publications underwent full‐text review, after removal of duplicates and retrieval of articles (Figure 1). Of these, a majority were removed because studies focused on identifying a health disparity in the study population but did not include an intervention for addressing cancer‐related health equity or a method for addressing cancer‐related health equity. In total, 40 articles were included after full‐text review (Table 1).

**TABLE 1:** Extracted Data from the Identified Publications.

Author, year	Purpose	Design/Sample	Primary Outcome/Metric	Major Findings
Screening interventions for cancer‐related health equity				
Arnold et al.^45^	Compare the effectiveness of two follow‐up approaches to a health literacy intervention to improve colorectal cancer screening: automated telephone call or personal call	620 participants aged 50–75 from four rural communities	Rate of colorectal cancer screening (Fecal immunochemistry test)	The overall FIT completion rate was 68% with no difference in the effectiveness of the follow‐up call method with both increasing the return rate by 9%
Bastani et al.^50^	Test a stepped intervention consisting of ethnically targeted and individually tailored print materials with follow‐up telephone counseling for those unscreened at 6 months, on colorectal cancer screening among an ethnically diverse sample of first‐degree relatives of colorectal cancer cases	1280 participants were relatives not current on colorectal cancer screening randomized to the intervention or usual care control arms	Rate of colorectal cancer screening	The cumulative print plus telephone intervention led to increased rate of colorectal cancer screening (26%) compared to usual care (18%). The print alone v. cumulative was not significantly different and was effective for Asian, Latino, and White but not African American participants
Brown et al.^55^	Assess a citywide public health campaign promoting screening colonoscopy	Cross‐sectional analysis of New York Cancer Registry, Vital Statistics, and Community Health Survey data	Colorectal cancer incidence, mortality, and colonoscopy rates	While overall colorectal cancer (CRC) incidence and mortality declined, incidence and mortality were highest among Blacks with mortality varying by neighborhood and associated with risk factors and access to care
Byrd et al.^24^	Determine the effectiveness of a lay health worker‐delivered intervention, AMIGAS, to increase Pap test screening among women of Mexican origin	613 women of Mexican origin were randomized to receive (1) full AMIGAS program (*n* = 151), AMIGAS without the video (*n* = 154), (3) AMIGAS without the flipchart (*n* = 155), or (4) usual care (*n* = 153)	Rate of cervical cancer screening (Papanicolaou test)	Women who participated in any of the intervention arms were significantly more likely to receive Pap testing; in the per‐protocol analysis, testing in the intervention groups (62%) was higher than usual care (29%, *p* < 0.001)
Screening interventions for cancer‐related health equity				
Cole et al.^51^	This RCT tested the effectiveness of a preclinical telephone‐based patient navigation intervention to encourage colorectal cancer screening among older Black men recruited from barbershops	731 Black men aged 50 years or older who were not up‐to‐date on colorectal cancer screening were randomized to (1) patient navigation by a community health worker for CRC screening, (2) motivation interviewing for blood pressure control by a trained counselor, or (3) both interventions	Rate of colorectal cancer screening	Participants were significantly more likely than those in the motivation interviewing only group to receive CRC screening at 6 months (17.5% in the patient navigation only group, 17.8% in the group that received both interventions, 8.4% in the motivational interviewing only group)
Cuaresma et al.^53^	Explored whether a lay health educator can increase colorectal screening among Filipino Americans ages 50–75 in Hawai'i	Cluster randomized controlled trial included 304 participants (77% women) randomized to lay health educator intervention or attention control group	Rate of colorectal cancer screening	The intervention group had a significant increase in the report of having received colorectal cancer screening (80% v 89%) following intervention but not in the control group
Dietrich et al.^44^	To assess the effect of telephone outreach (support and education) on colorectal cancer screening	2240 women in Medicaid managed care organizations who were over‐due for colorectal cancer screening	Rate of colorectal cancer screening	Telephone outreach delivered by staff increased colorectal cancer screening by 6% more than usual care and by 15.1% more than usual care among over‐due women reached by the intervention
Galiatsatos et al.^19^	To evaluate the effect of a dedicated Tobacco Treatment Clinic offering evidence‐based strategies for smoking cessation and lung cancer screening	92 patients aged 50 years and older having a minimum 20 pack‐year smoking history	Rate of lung cancer screening with low‐dose chest tomography	Of the 92 patients, 68 (73.9%) had lung cancer screening with 51 receiving their first lung cancer screening scan
Screening interventions for cancer‐related health equity				
Goldman et al.^48^	To evaluate an outreach intervention (mailed fecal immunochemical test (FIT) kit with text message 2 days later, followed by automated phone call and text message 2 weeks later, followed by navigator phone call at 3 months) on colorectal cancer screening compared to usual care	420 patients who had never completed colorectal cancer screening	Rate of colorectal cancer screening (fecal immunochemistry test)	Outreach led to higher likelihood of completing FIT testing compared to usual care (36.7% v 14.8%, *p* < 0.001) with FIT completion higher among patients with increased clinical visits
Green et al.^56^	Examine whether guaranteed money or probabilistic lottery financial incentives conditional on completion of colorectal cancer screening increase screening uptake.	838 participants randomized to (1) mailing that included information of CRC screening and test choices, FIT test and reminder letter (*n* = 284), (2) mail and monetary incentive of $10 on FIT completion (*n* = 270) or (3) mail and lottery with 1 in 10 chance of receiving $50 on FIT completion	Rate of colorectal cancer screening (fecal immunochemistry test)	There was no significant increase in CRC screening overall but the mail and monetary and mail and lottery interventions increased FIT completion (7.7%, 7.1%) compared to mail only group. These interventions increased FIT completion more among patients with Medicaid insurance
Hendren et al.^46^	Assess a multimodal intervention (letters, automated phone calls, prompts, and mailed fecal immunochemical testing (FIT) kit) on cancer screening among patients in a safety‐net primary care practice	366 average‐risk patients over‐due for mammography or colorectal cancer screening randomly assigned to intervention (*n* = 185) or usual care (*n* = 181)	Rate of cancer screening (mammography or FIT) at 1 year post‐intervention	Significantly higher rates of cancer screening were obtained in the intervention group for mammography (29.7% vs 16.7%, *p* = 0.034) and colorectal cancer screening (37.7% v 16.7%, *p* = 0.0002)
Screening interventions for cancer‐related health equity				
Hirko et al.^47^	To determine the effect of a mailed motivational messaging screening reminder letter with option to call and request a free at‐home fecal immunochemical screening test (intervention) or standard letter reminding screening due (control)	7812 adults age 50–75 years old due for colorectal cancer screening in a rural health system randomly assigned to the intervention (*n* = 3906) or control (*n* = 3906) group	Rate of colorectal cancer screening within 6 months after mailed invitation	The mailed motivation messaging letter with a low‐cost screening alternative improved screening (30.1%) compared to control (22.5%, *p* < 0.001). Intervention participants have 49% higher odds of being screened compared to control (OR = 1.49, 95% CI‐1.34, 1.65)
Horne et al.^52^	Investigate the effect of patient navigation on increasing colorectal cancer screening among older African Americans	1220 adults were randomized to the control group receiving printed education materials or intervention that received a patient navigator plus printed materials	Rate of colorectal cancer screening	The intervention group was more likely to report receipt of colorectal cancer screening (72.5%) compared to control (58.6%, *p* < 0.04), with stronger effects of navigation among participants 65–69 years old with an adequate health literacy level
Huf et al.^49^	Compare the effectiveness of a single text message outreach to serial text messaging and mailed fecal home test kits on colorectal cancer screening rates.	440 participants (mean age 57.4 years), 63.4% women, 87.7% Black, 96.1% spoke English as first language, 19.1% uninsured randomized to intervention (*n* = 220) or control (*n* = 220) arm	Rate of colorectal cancer screening (colonoscopy or FIT) at 12 weeks	At 12 weeks, there was an absolute 17.3% point increase in colorectal cancer screening in the intervention arm (19.6%) compared to the control arm (2.3%); serial text messaging with opt‐out mailed FIT kit outreach improved screening rates
Jandorf et al.^54^	Investigate the impact of professional or community‐based peer navigators on rate of colorectal cancer screening colonoscopy	240 African American patients referred for screening colonoscopy at a primary care clinic with a direct endoscopic referral system	Rate of colorectal cancer screening	Professional and community‐based peer navigation resulted in 80.0% and 71.3% screening rates and both groups reported high levels of satisfaction and trust
Screening interventions for cancer‐related health equity				
Lucas et al.^57^	Examine how standard and culturally targeted versions of gain and loss‐framed messaging affect African American colorectal cancer (CRC) receptivity and behavior	457 African Americans viewed an informational video about CRC screening and were randomized to receive a gain‐ or loss‐framed message about screening	Rate of colorectal cancer screening	Participants were more receptive to CRC screening if loss‐framing was culturally targeted and reduced anticipatory racism more than standard loss‐framing. However, there was no difference in CRC screening uptake between groups
Ma et al.^25^	Evaluate the effectiveness of a multifaceted and culturally appropriate intervention in increasing Pap testing rates in Vietnamese American women	30 Vietnamese community organizations were randomized to either intervention or control condition; 1416 participants were followed. Intervention consisted of group‐based education by community health educators, multimedia educational materials in Vietnamese and reminders	Rate of cervical cancer screening (Papanicolaou test)	Intervention group participants had a significantly higher rate of Pap testing (60.1%) compared to the control group (1.6%, *p* < 0.0001)
Miller et al.^26^	Explore the impact of a tailored telephone counseling intervention on increasing follow‐up adherence after an abnormal Pap smear result among low‐income, minority women	211 women were randomly assigned to receive (1) telephone counseling tailored to the barriers elicitied, (2) barriers assessment and mailed home tailored barriers print brochure, or (3) enhanced standard care with barriers assessment; all received telephone reminders	Rate of initial colposcopy	Telephone counseling tailored to the barriers elicited had significant impact on adherence to follow‐up recommendation compared to the other intervention (*p* < 0.05) and was more effective among women with a high school education or less
Screening interventions for cancer‐related health equity				
Molina et al.^20^	Assess differences in educate+LA addressing participants' breast cancer prevention and screening behavior with empower+LA which addressed participants' and their social networks' breast cancer screening	145 Latinas non‐adherent to USPSTF breast cancer screening guidelines	Rate of breast cancer screening (mammography)	Empower+LA participants were more likely to report obtaining mammograms (72% vs 48%), engaging more individuals about breast cancer, initiating breast cancer conversations and mammography
Murphy et al.^27^	To compare the relative efficacy of a fictional narrative film to a more traditional nonnarrative film promoting cervical cancer screening	704 women aged 25 to 45 years were randomly assigned to view either a narrative or nonnarrative film	Rate of cervical cancer screening (Papanicolaou test)	Both films increased cancer‐related knowledge (*p* < 0.001), attitudes (*p* = −0.007) and receipt of a Pap test (*p* = 0.05) but the narrative had a larger effect especially for Mexican American women
Rapkin et al.^28^	To investigate the effects of a cancer screening and prevention community‐based participatory research partnership (HealthLink) program delivered in local libraries	9374 respondents were interviewed in Queens, New York	Rate of colorectal, breast and cervical cancer screening, smoking quit attempts, and smoking cessation	Increased colorectal, breast, and cervical cancer screening, and smoking quite attempts were associated with program implementation; increases were strongest among respondents born outside the United States or least engaged in care
Schwartz et al.^21^	This cohort study examined whether providing individualized breast cancer risk estimates is associated with an increased rate of screening mammography	347 women aged 25 to 69 years without a personal history of breast cancer presenting for an annual visit primary care clinician	Rate of breast cancer screening (mammography)	Of the 347 women, 188 were age‐eligible for mammography. Among women of racial and ethnic minority groups with high‐risk for breast cancer, there was an increase in screening mammography from 37% during usual care to 51% following risk assessment
Screening interventions for cancer‐related health equity				
Sinicrope et al.^22^	Develop and evaluate a mammography intervention that provides hope about cancer prevention and treatment.	25 participants were randomized to the intervention (*n* = 13) or control group (*n* = 12)	Breast cancer screening (mammography) at 3‐month post‐intervention	There was no difference between groups but the rate of mammography (44%) was higher among women with a support person (57%) compared to those without (27%)
Studts et al.^30^	Test the effectiveness of a faith‐placed lay health advisor intervention to increase cervical cancer screening among middle‐aged and older women with low screening rates	345 women randomized to treatment (*n* = 176) or wait‐list control group (*n* = 169). Intervention provided lay health advisor home visits and newsletters addressing barriers to screening	Cervical cancer screening (Papanicolaou test)	Treatment group participants (17.6% screened) had over twice the odds of reporting Pap test receipt compared to the wait‐list control group (11.2%, *p* = 0.04)
Thompson et al.^29^	Investigate two approaches to increase cervical cancer screening among Latinas enrolled in a federally qualified health center	443 Latinas were randomized to (1) receive a video intervention delivered to their home (*n* = 150), (2) video plus home‐based educational session (*n* = 146) or (3) usual care (*n* = 147)	Cervical cancer screening (Papanicolaou test)	Women who received the video plus home‐based education session had higher rate of cervical cancer screening (53.4%) compared to video only (38.7%) or usual care (34.0%) (*p* < 0.01)
Wong et al.^23^	Investigate the impact of a same‐day breast biopsy program for patients with serious mental illness and public payer‐insured patients <65 years of age versus women without serious mental illness	2026 biopsies were performed pre‐intervention and 2361 biopsies post‐intervention	Days from breast biopsy recommendation to completion and proportion of biopsies performed as same‐day biopsy	After routinely offering same day biopsy for patients with serious mental illness the proportion increased from 15.3% to 36.09% (p < 0.001), with no evidence of disparities in the women with serious mental illness on public payer insurance
Treatment interventions to improve cancer‐related health equity				
Cykert et al.^31^	To evaluate a system‐based intervention consisting of (1) real‐time warning system derived from electronic health records (on missed appointments/tests/procedures); (2) race‐specific feedback to clinical teams on treatment completion rates, and (3) a nurse navigator	2841 early stage lung cancer patients (16% Black) in the retrospective group; 360 (32% Black) in the intervention group. All consented participants received the intervention	Receipt of potentially curative treatment (surgical resection or stereotactic radiation)	Crude treatment rates were 78% for White patients v 69% for Black patients in the retrospective group; within the intervention cohort the crude rate was 96.5% for Black v 95% for White patients
Dessources et al.^32^	To examine the effects of a socially determined cervical cancer care navigation program at a public safety‐net hospital to improve treatment adherence	46 women with stage IB1 to IVA cervical cancer who underwent primary chemoradiotherapy were prospectively enrolled in the navigation program and compared to 84 women treated previously without navigation	≥5 cycles of cisplatin chemosensitization; Initiation of brachytherapy during external beam radiotherapy; Completion of external beam radiotherapy and brachytherapy Primary chemoradiotherapy completion with ≤63 days; Median duration of primary chemoradiotherapy	The percentage of patients receiving ≥5 cycles of weekly cisplatin increased from 74% to 93% (*p* < 0.01) and rates of the initiation of brachytherapy during external beam radiotherapy increased from 49% to 78% (P < 0.01). The median treatment time was reduced from 67 days in the nonnavigated patients to 55 days in the navigated patients (*p* < 0.01). Approximately 95% of navigated patients who completed pCRT did so within 63 days, compared with 52% of non‐navigated patients (*p* < 0.01). Treatment completion within 63 days was associated with significantly improved overall survival
Treatment interventions to improve cancer‐related health equity				
Durand et al.^37^	To compare the influence of an option grid or picture option grid conversation aid with usual care on decision quality, treatment choice, treatment intention, shared decision‐making, anxiety, quality of life, decision regret, and coordination of care among women with breast cancer	571 women with newly diagnosed breast cancer	Decision quality, treatment choice, treatment intention, shared decision‐making, anxiety, quality of life, decision regret, coordination of care	The picture option grid (*n* = 248) arm had higher knowledge, improved decision process, lower decision regret and more shared decision‐making compared to usual care, and had more impact among women with lower socioeconomic and health literacy. The option grid (*n* = 66) arm had higher decision process, better coordination of care, and more shared decision‐making compared to usual care. Neither intervention affected concordance, treatment choice, or anxiety
Nattinger et al.^35^	Examine whether a policy precluding payment for breast cancer surgery for New York Medicaid beneficiaries undergoing surgery in low‐volume facilities led to reduce socioeconomic disparities in mortality	14,183 Medicare beneficiaries with breast cancer in 2006–2008 or 2014–2015 after policy implementation	All‐cause mortality at 3 years after diagnosis	Low socioeconomic and Medicare‐only patients had better 3‐year survival after policy implementation. The decline in mortality was larger in magnitude for low socioeconomic women than others with a 53% smaller socioeconomic survival disparity after the policy when adjusting for age, race, and comorbid illness
Treatment interventions to improve cancer‐related health equity				
Nipp et al.^36^	An interrupted time series design evaluated implementation of a cancer care equity program (CCEP) to address financial burden associated with clinical trial participation, specifically costs of travel and lodging	1217 patients, age 18 and older, who had enrolled in cancer clinical trials at the institution after cancer care equity program implementation were assessed according to medical record review	Rate of cancer clinical trial enrollment	Enrollment in clinical trials increased significantly after CCEP implementation with a greater proportion of CCEP participants being younger, female, in phase I trials, lived further away, had lower incomes, and had metastatic disease
Swayze et al.^33^	To compare management strategies, outcomes and access to care for patients in a small city and surrounding rural communities before and after establishing a full‐time gynecologic oncology office	381 women diagnosed with ovarian cancer, with 171 diagnosed prior to establishing the gynecologic oncology office	Surgery by a gynecologic oncology specialist, receipt of surgery locally, complete lymph node dissection, receipt of chemotherapy, median survival time	Women receiving care at the local gynecologic oncology office were more likely to undergo surgery by a specialist (97.1% v 53.2%), receive surgery locally (79% v 43.3%), undergo complete lymph node dissection (63.3% v 38.6%) and receipt of chemotherapy increased from 10.3% to 76.9% (all *p* < 0.01). Median survival time increased from 30.8 months to 52.5 months and travel decreased from a mean of 47.9 miles to 26.8 miles
Tang et al.^34^	To investigate the impact of a multidisciplinary breast program of timeliness of care at a safety‐net hospital	102 women with breast cancer diagnosis	Time to completion of imaging, biopsy and initial treatment	There was no difference in delivery of screening, work‐up, and treatment by race of women within the same socioeconomic status
Interventions for cancer survivors and family caregivers				
Dionne‐Odom et al.^39^	To assess the feasibility, acceptability, and potential efficacy of a lay navigator‐led early palliative care telehealth intervention for African American/Black and or rural‐dwelling family caregivers of individuals with advanced cancer.	63 family caregivers of patients with newly diagnosed stage III/IV solid tumor cancers randomized to receive the intervention (*n* = 31) or usual care (*n* = 32)	Caregiver distress and quality of life	Recommending the program to another caregiver was rated on average as 9.4 (out of 10). At 24 weeks, the distress score and quality of life improved (0.3 ± 1.44; 0.4 ± 4.5 points, respectively) in the intervention group and worsened (1.99 ± 1.39; 1.1 ± 4.3 points) in the control group
Hirko et al.^38^	This quasi‐experimental study evaluated the implementation of a clinic‐based physical activity program for cancer survivors at a rural community oncology setting	24 cancer survivors residing in a rural setting	Improvement in cancer symptoms and quality of life	Only 0.59% of cancer survivors participated; however, there were improvements in fatigue (*p* = 0.03), constipation (*p* = 0.02), pain (*p* = 0.007), and sleep quality (*p* = 0.008) from pre‐ to post‐intervention
Methods to evaluate cancer‐related health equity				
Biddell et al.^43^	A national sample of patients with cancer employed at diagnosis was conducted to assess the impact of cancer diagnosis and treatment on employment	619 adults with cancer diagnosis	Income loss and changes in health insurance coverage	Over 83% reported taking significant time off from work during cancer diagnosis and treatment, with 64% having substantial income loss and 31% changing insurance coverage; Non‐Hispanic Black and Hispanic or Latinx patients had a 10.2% and 12.4% higher probability of substantial income loss and 9.3% and 10.0% higher probability of changes in health insurance compared to Non‐Hispanic White patients
Chapman et al.^58^	To compare tradeoffs of breast cancer screening strategies for Black women versus White women under current guidelines	A 1980 U.S. birth cohort of Black and White women	Outcomes included benefits (life‐years gained, breast cancer deaths averted, and mortality reduction), harms (mammographies, false positives, and overdiagnosis), and benefit‐harm ratios (tradeoffs) by race. Efficiency, mortality disparity reduction, and equity in tradeoffs were evaluated	The single model demonstrated that initiating biennial screening in Black women at age 40 years reduces breast cancer mortality disparities and has benefit‐harm ratios similar to tradeoffs of White women screened biennially from ages 50 to 74 years
Methods to evaluate cancer‐related health equity				
Li et al.^40^	Determine whether Medicaid expansion impacted racially more diverse states similarly as racially less diverse states in endocrine therapy prescriptions.	State Medicaid expansion and racial diversity status were used to study Medicaid‐financed endocrine therapy prescriptions from 2011 to 2018 from the Medicaid State Drug Utilization Database	Rate of endocrine therapy prescription	Endocrine therapy prescriptions increased sharply in expansion states compared to non‐expansion states (*p* = 0.057) and was sharper for racially more diverse expansion states (*p* = 0.008). Gaps in ET prescriptions between expansion and nonexpansion states with racially more diverse populations widened
Michel et al.^41^	This case–control study examined the impact of Medicaid expansion on diagnosis and management of genitourinary cancers	340,552 patients with newly diagnosed genitourinary cancer in the National Cancer Database from 2011 to 2016	Diagnosis of early‐stage kidney and prostate cancers	Compared with states that did not expand Medicaid, Medicaid expansion was significantly associated with decreased uninsured rate, increased early‐stage diagnosis, and increased proportion of patients receiving active surveillance for low‐risk prostate cancer, with larger magnitudes of association observed in low‐income populations
Ritzwoller et al.^42^	This cohort study estimated the population level changes associated with the 2021 USPSTF expansion of lung cancer screening eligibility by sex, race and ethnicity, sociodemographic factors and comorbidities in 5 community‐based healthcare systems	Data from 34,528 adults age 50–80 years who currently or previously smoked and had electronic health record data that captured pack‐year and quit‐date information	Increase in eligibility for lung cancer screening	The 2021 USPSTF recommendations expanded eligibility to 18,533 individuals (53.7% increase) and included a larger proportion of women and more racial or ethnic minority groups

Of these 40 articles, the majority addressed cancer screening, with most (*n* = 13) focusing on reducing inequities in colorectal cancer (CRC) screening. One article addressed lung cancer screening,^19^ four articles addressed breast cancer screening with mammography^20^, ^21^, ^22^ or same‐day biopsy.^23^ Seven articles addressed cervical cancer screening^24^, ^25^, ^26^, ^27^, ^28^, ^29^ or follow‐up colposcopy.^30^

Eight articles described interventions focused on improving cancer‐related health equity. Several studies (*n* = 6) included a metric pertaining to receipt of standard care^31^, ^32^, ^33^, ^34^ or survival duration/mortality.^33^, ^35^ One study evaluated the rate of clinical trial enrollment^36^ while another evaluated shared decision‐making, decision regret, and quality of life.^37^

Two articles included cancer survivors or family caregivers, using interventions focused on improving physical activity for cancer‐related symptoms and quality of life^38^ or telehealth support for family members in palliative care to reduce caregiver distress and increase quality of life.^39^ Other studies (*n* = 4) investigated methods for addressing cancer‐related health equity through expansion of Medicare eligibility,^40^, ^41^ implementation of current screening guidelines or the implementation of recommendations from the U.S. Preventative Services Task Force for lung cancer screening.^42^ One article studied the impact of cancer diagnosis and treatment on employment income loss and health insurance.^43^

### 3.1 Strategies for improving equity in cancer screening

Across the studies we reviewed, efforts to improve equity of screening rates in different populations with cancer (especially colorectal cancer (CRC) screening among rural and Black/African American participants) were a major focus. These studies demonstrated that outreach using mailings, telephone, or text messaging to remind and encourage completion of CRC screening can be effective across populations.

Consistent with these prior studies, results from the current review suggest that telephone outreach providing support and education can improve CRC screening return among women in Medicaid‐managed care organizations.^44^ Arnold et al.^45^ found that follow‐up phone calls (either automated or personal) following mail return of fecal immunochemistry test (FIT) kits were associated with improved screening returns among rural residents. Use of mailings and automated phone calls was also associated with increased CRC screening rates for participants in a safety‐net primary care practice,^46^ as well as rural residents.^47^ Goldman et al.^48^ also found that use of text messages and automated phone calls, combined with a personal call from a patient navigator for those overdue for cancer screening at 3‐months, increased FIT return rates compared to usual care. Use of serial text messages versus a single text message was 17.3% more effective for improving FIT return rates among a largely Black/African American sample.^49^

Other strategies for increasing equity in cancer screening included culturally sensitive educational materials paired with a lay navigator or community educator. Bastani et al.^50^ tested whether pairing telephone counseling with ethnically targeted print materials tailored to individual's unique risk factors could improve CRC screening rates among those unscreened at 6 months. Results found that the pairing intervention was effective for improving CRC rates in Asian, Latinx, and White, but not Black/African American participants. Similarly, Cole et al.^51^ tested whether adding telephone‐based patient navigation to motivational interviewing improved CRC screening among older Black men,^51^ finding both navigation alone and adding navigation to motivational interviewing was superior to motivational interviewing alone. Other studies demonstrated that the use of patient navigation was also more effective than printed materials for improving CRC screening among older Black men.^52^ Use of lay health educators was also effective in improving CRC screening rates among Filipino Americans,^53^ and both professional and community‐based peer navigators were effective at improving CRC screening among Black/African Americans.^54^

While efforts to promote CRC screening using community‐based interventions were associated with improvements in outcomes, the degree to which community‐based interventions improved equity for different racial and ethnic groups were mixed. Brown et al.^55^ evaluated the impact of a citywide public health campaign for promoting screening colonoscopy and found that while overall incidence and mortality related to colorectal cancer declined, Black residents continued to have higher incidence and mortality rates. Conversely, a community‐based cancer screening program delivered through local libraries was associated with higher CRC screening, especially among respondents born outside the United States and those who were least engaged in care.^28^ Other information or incentive‐based strategies have been tested with limited efficacy.^56^, ^57^

Ten articles highlighted the importance of individualized, culturally appropriate outreach strategies and/or involving social support resources (interpersonal or network) for improving cancer screening rates among minority populations. Cervical cancer screening rates improved with a lay health worker‐delivered intervention called AMIGAS among women of Mexican origin^24^ and a multifaceted and culturally appropriate group‐based intervention among Vietnamese American women.^25^ Other culturally sensitive interventions that provided more social interaction with participants were also effective in improving screening rates.^27^, ^29^, ^30^ While mailings and automated phone calls appeared to be effective in improving overall breast cancer screening rates,^46^ use of individualized risk assessment was effective in improving mammography rates among racial and ethnic minority groups at higher risk for breast cancer.^21^ However, breast cancer screening rates were more likely to improve when a support person was identified among Native American women^22^ or when engaging Latinas personal and social breast cancer screening network compared to only addressing personal risk factors.^20^ Conversely, tailored telephone counseling had a significant effect on adherence to follow‐up recommendations after an abnormal Pap test compared to mailed home materials or standard care among low‐income women.^26^

### 3.2 Treatment interventions to improve cancer‐related health equity

While efforts to increase equity in cancer screening between groups facing disparities were a major focus among articles we reviewed, fewer articles focused on interventions aimed at improving equity in treatment, with several notable exceptions. Cykett et al.^31^ used a pragmatic design to evaluate the impact of providing clinicians real‐time warnings about missed appointments and procedures using the electronic health record (EHR), race‐specific feedback on completion rates, and follow‐up/intervention by a nurse navigator. Results found that the intervention was associated with reduction in disparities in receipt for curative treatment among Black/African American patients.

Similarly, a lay navigation program at a public safety‐net hospital for women with cervical cancer was associated with an almost 20% increase in receipt of curative treatment with chemoradiotherapy among participants.^32^ However, results were not universal; evaluation of a multidisciplinary breast program on timeliness of care from diagnosis to treatment found the program was not impactful in improving delivery of screening, work‐up, and treatment for women due to inadequate infrastructure and support.^34^

Nattinger et al.^35^ examined the effect of ending a policy that precluded payment for breast cancer surgery among Medicaid beneficiaries in New York. Results found evidence of improved 3‐year survival and a 53% reduction in socioeconomic disparity during implementation of the policy. In addition, use of a cancer care equity program designed to mitigate the financial burdens associated with transportation and lodging for participants in clinical trials led to a significant increase in clinical trial enrollment, including among females and individuals who were younger, living further away, living with metastatic disease, and with lower incomes.^36^ In 2021, Swayze et al.^21^ evaluated the impact of establishing a local gynecologic oncology office on access to care, guideline‐concordant care, and median survival among women with cervical cancer. Results showed that women receiving care locally were more likely to undergo guideline‐concordant care, with an increase in median survival from 30.8 to 52.5 months. Likewise, Durand et al.^37^ compared different decision support tools for patients with breast cancer, finding that a picture option grid led to higher knowledge, improved decision process, lower decision regret and more shared decision‐making compared to usual care, especially among women with lower socioeconomic status and health literacy scores. However, the interventions did not affect concordance of care, treatment choice, or levels of patient‐reported anxiety.

### 3.3 Interventions addressing equity in cancer survivors and caregivers

Only two of the 40 studies reviewed described interventions designed to improve equity in outcomes for cancer survivors or family caregivers. Hirko et al.^38^ evaluated the impact of a clinic‐based physical activity program for rural‐residing cancer survivors. While only 0.59% of the eligible cancer survivors participated, the intervention was associated with improvements in fatigue, constipation, pain, and sleep quality post‐intervention. Similarly, Dionne‐Odom et al.^39^ assessed the effectiveness of a lay navigator‐led early palliative care telehealth intervention for Black/African American and/or rural‐dwelling family caregivers of individuals with advanced cancer. The intervention was found to be feasible, acceptable, and was associated with statistically significant reductions in distress and higher quality of life scores compared to the control group.

### 3.4 Methods to evaluate cancer‐related health equity

Of the five studies employing methods for evaluating health equity, four examined the effect of policy level changes, including adoption of cancer screening recommendations from the U.S. Preventative Task Force (USPTF) and Medicaid expansion. Chapman et al.^58^ studied a 1980 U.S. birth cohort of Black and White women, comparing tradeoffs of breast cancer screening strategies between groups. Results found that initiating biennial screening in Black women at age 40 could reduce disparities in breast cancer mortality with benefit‐harm ratios similar to White women ages 50 to 74 years old who are screened biennially. Likewise, Ritzwoller et al.^42^ examined the impact of the 2021 USPTF expansion of lung cancer screening eligibility, finding that the expansion led to a 53.7% increase in lung cancer screening eligibility across five community‐based healthcare systems. Of note, under the expanded eligibility criteria, a larger proportion of women and more racial and ethnic minority groups were eligible for lung cancer screening, underscoring the potential impact of policy‐level changes in efforts to reduce disparities in cancer‐related outcomes.

Similarly, when Li, Najarian & Halpern^40^ examined whether Medicaid expansion influenced endocrine therapy prescriptions used to treat breast cancer in racially more diverse and less diverse states, they found that Medicaid expansion increased the number of endocrine therapy prescriptions filled, particularly in racially more diverse expansion states. Another study found that Medicaid expansion was associated with decreased uninsurance, increased early‐stage diagnosis of genitourinary cancers, and increased active surveillance for low‐risk prostate cancers, especially among low‐income populations. Biddell et al.^43^ examined the impact of cancer diagnosis and treatment on employment income and health insurance in a national sample of adults. They found that Non‐Hispanic Black and Latinx patients had a 10.2% and 12.4% higher probability of substantial income loss, and a 9.3% and 10.0% higher probability of changes in health insurance compared to Non‐Hispanic White patients.

## 4 DISCUSSION

The purpose of this scoping review was to identify cancer‐specific health equity interventions and metrics that have been deployed in practice, to examine how these indicators have been implemented and evaluated across the cancer care continuum, and to identify current research gaps in the field. Results of the review led to several key findings and identified gaps in the field, which are discussed below.

First, the studies included in the review demonstrate that a major focus for the field related to equity has been cancer screening. Results suggest that use of strategies designed to improve outreach and follow‐up can positively improve cancer screening rates (especially, CRC screening rates), and—critically—that these approaches may be effective for improving screening rates in populations disproportionately impacted by cancer such as Black/African Americans and rural Americans.^26^, ^45^, ^46^, ^48^, ^49^, ^50^ With advances in technology and home‐based screening methods, it is incumbent for healthcare systems and insurers to facilitate more accessible options for cancer screening, especially for individuals with known risk factors and hourly workers who are greatly impacted by taking time off work for screening appointments.

Second, the review findings underscore the importance of using community engagement to build support for cancer screening in communities facing systematic inequalities.^20^, ^28^, ^51^, ^52^, ^53^, ^54^, ^55^ Overall, outreach by professional healthcare providers and lay educators alike appears to improve screening rates, including minority groups.^25^, ^29^, ^30^ In addition, results suggest that strategies designed to increase access to cancer screening can reduce disparities in guideline‐concordant screening.^19^, ^23^ However, additional evidence is needed to clarify which strategies are likely to be most effective for building engagement in different communities.

Third, and closely related, access to patient navigation during screening and treatment can positively impact cancer‐related health equity.^31^, ^32^ Use of technology to identify patients who may benefit from navigation^31^ as well as policies to reduce financial burden associated with cancer treatment may positively influence timeliness in receiving treatment and reduce disparities in mortality.^35^ Of note, Patel and colleagues^59^, ^60^, ^61^ have an ongoing study that tests an intervention designed to improve advance care planning and symptom management among low‐income and minority hourly‐wage workers with cancer by providing a lay health worker to all patients newly diagnosed with cancer. The intervention aims to educate and activate patients to engage in advance care planning and symptom management with their oncology providers and will assess quality of life, patient activation and satisfaction with decision‐making, symptom burden and total healthcare use and costs.

Findings from the scoping review also suggest that strategies designed to increase access to cancer specialists and resources needed to participate in cancer clinical trials (including travel and lodging) can help reduce disparities in receipt of guideline‐concordant care and clinical trial participation. In addition, the results support the effectiveness of health services designed for cancer survivors and family caregivers on reducing disparities in cancer‐related symptoms and caregiver distress.^38^, ^39^

A notable finding was the importance of evaluating the impact of policy changes on both cancer outcomes and equity. Several studies found that policy changes were associated with reductions in disparities.^40^, ^41^, ^58^ Ritzwoller et al.^42^ evaluation of the USPTF expansion for lung cancer screening eligibility provides actionable steps to inform the community so that newly eligible patients are aware and can access screening. The financial impact of cancer diagnosis and treatment creates a widening disparity that requires a multifaceted approach.^43^ Mohan and Chattopadhyay^11^ conducted a systematic review of economic evaluations of interventions leveraging social determinants of health, such as patient navigators, transportation assistance, and home‐testing, to improve screening for breast, cervical and colorectal cancer. Improved cancer screening for underserved communities was cost effective with a median intervention cost of $123.87, a median incremental cost per additional person screened of $250.37, and a median incremental cost per quality‐adjusted life‐year (QALY) of $3120.00.

Finally, results from this scoping review highlight the lack of clarity about how best to measure equity (or a lack thereof) in the various dimensions of the cancer continuum. While it is clear that equity has been a consistent focus for the field for decades, studies evaluating interventions specifically designed to address equity, or to evaluate the degree to which certain outcomes can serve as reliable “equity metrics” in the cancer space are lacking. The lack of generalizable equity metrics in the oncology space underscores both the *contextual* and often *layered* nature of barriers that can prevent individuals from getting equitable access to prevention, screening, diagnosis, treatment, and survivorship care for cancer. Adding to this challenge, it could be argued that, in certain populations, metrics such as knowledge of CRC and intent to screen could be considered equity metrics, although the degree to which these endpoints directly measure equity, or merely act as mirrors reflecting deeper issues of equity, remains to be determined.

### 4.1 Gaps in cancer‐related health equity research

While this scoping review identified studies across the cancer care continuum, approximately 20% of the studies only incorporated one level of intervention for addressing health disparities, such as outreach and follow‐up calls. Addressing other social determinants of health and structural barriers, including cancer services in local communities, receipt of guideline‐concordant care, reduced or free screening and treatment, and living wages or time‐off to undergo screening and treatment are critical areas that have yet to be studied. Evidence to inform the most effective strategies for building engagement in different communities and in using patient‐reported outcomes or other sources of data advance equity are also needed. In addition, all of the identified studies were conducted in adult populations, lacking participants who were children or families with children. It has been estimated that scale‐up of interventions for improving access to healthcare services and childhood cancer treatment could not only avert childhood deaths from cancer but would produce a net global lifetime productivity gain.^62^ Studies to address the structural barriers of underserved populations, childhood cancer disparities and the impact on the family unit are especially needed to advance equity across the cancer care continuum.

### 4.2 Limitations

The scoping review was focused on cancer‐specific health equity metrics in the United States of America, and results of the review should only be generalized in this context. As a scoping review, the approach to the literature search was systematic but not exhaustive, and other interventional cancer studies may not have been included. Bias may have influenced the interpretation of findings and identification of cancer‐related health equity metrics that have been previously studied and or tested due to the search strategy, only including research studies published in the English language, conducted in the United States of America, and within the specific time frame. While the scoping review only provides a snap shot of important work in the field of cancer‐related health equity metrics, the intention was to highlight crucial gaps and strategies to advance future research in the United States of America.

### 4.3 Conclusion

This scoping review identified interventions that were effective in reducing cancer‐related disparities and health equity metrics that may be useful for future studies to incorporate. Interventions that were found to be effective in reducing disparities included patient reminders, access to patient navigators and local services, culturally sensitive outreach and support resources (i.e., social support, transportation), and policies to expand covered services for cancer screening and treatment. Evidence to inform the most effective strategies for building community engagement in cancer screening as well as the use of patient‐reported outcomes and other sources of date to evaluate cancer‐related outcomes is needed. Cancer‐related health equity metrics used to evaluate whether the interventions were associated with more equitable outcomes included traditional measures (i.e., rate of screening or morbidity and mortality) as well as more nuanced metrics such as the rate of early‐stage versus late‐stage diagnosis, receipt of guideline‐ and goal‐concordant care, and financial burden of cancer screening and treatment. A majority of the research studies included in the scoping review were focused on cancer screening, suggesting that more interventional research is needed to advance equity in treatment and survivorship outcomes, especially among underserved populations. Finally, interventions developed to address the social and structural barriers of low‐resourced communities, caregivers, and children or families with children are crucial for advancing health equity across the cancer care continuum.

## AUTHOR CONTRIBUTIONS

Angela Starkweather: Conceptualization‐lead, data curation‐equal, methodology‐equal, resources‐lead, writing—original draft‐lead, writing—review & editing‐equal. Bevin Cohen: Conceptualization‐supporting, data curation‐supporting, methodology‐supporting, writing—original draft‐equal, writing—review & editing‐equal. Tamryn Gray: Conceptualization‐supporting, methodology‐supporting, writing—original draft‐equal, writing—review & editing‐equal. Lauri Linder: Conceptualization‐equal, methodology‐equal, writing—original draft, writing—review & editing. Noah Zanville: Conceptualization‐equal, methodology‐equal, writing—original draft, writing—review & editing.

## FUNDING INFORMATION

No funding was received for this work. This research was supported (in whole or in part) by HCA Healthcare and/or an HCA Healthcare affiliated entity. The views expressed in this publication represent those of the authors and do not necessarily represent the official views of HCA Healthcare or any of its affiliated entities.

## CONFLICT OF INTEREST STATEMENT

The authors declare no conflicts of interest.

## Supporting information


Data S1.

## Data Availability

Data compiled for the literature review are available upon request.
