# Supplementary material for: Cancer‐Specific health equity metrics in the United States of America: A scoping review
Source: Cancer Med. 2023 Apr 5;12(10):11889–906. doi: 10.1002/cam4.5881 (PMC10242343; doi:10.1002/cam4.5881)
Supplement: Supplementary file 1 — Data S1. [file CAM4-12-11889-s001.docx]

**Supporting Information. Brief Timeline of National Efforts to Address Cancer-related Health Equity**

| **Year** | **National Efforts** |
| --- | --- |
| 1998 | The NCI framed efforts to address cancer care disparities within the quality aims of the Institute of Medicine (safety, effectiveness, patient-centeredness, timeliness, efficiency, equity) (Chassin & Galvin, 1998). |
| 2012 | To strengthen efforts toward equity, the NCI placed a 2012 requirement for NCI-designated cancer centers to define and address the needs of a local “catchment area”, which would in theory inform the initiatives of the cancer center in identifying disparities and take focused action to address them. |
| 2016 | Following this mandate, NCI required comprehensive NCI-designated cancer centers to have a Community Outreach and Engagement (COE) component in 2016. Both measures were designed to build systems and infrastructure within cancer centers to bolster efforts in identifying cancer-related health disparities and develop avenues for improving health equity across social groups (Polite et al., 2017). While these mandates are defined, implemented, and evaluated internally within each cancer center, the outcomes are folded into the ongoing NCI-designation review process. |
| 2020 | Following the development of an agenda for cancer research utilizing a multilevel framework, broad recommendations have been published on how to address determinants of health and improve cancer care outcomes, including:   - use of innovative screening processes, such as home self-sampling for HPV and/or cytology, incorporation of patient navigation, and changes to improve health care coverage (Temkin et al., 2018); - interventions to reduce social and structural inequities (e.g., income and wealth inequality, models of care that consider social risk, focused training for health care providers) (Alcaraz et al., 2020); - modification of institutional environments (e.g., access to high-quality care, enhancement of standards relevant to patient social circumstances); - improvement of living environments (e.g., enhancement of surveillance data to detect exposure to toxins, increased cross-sectoral collaboration) (Alcaraz et al., 2020); - involvement of insurance and payer systems, employer organizations and labor unions in reducing health disparities (Patel, Snyder, & Brawley, 2020); - community engagement, increasing the diversity of the healthcare workforce, and greater inclusion in clinical trials (Patel, Lopez et al., 2020; Siker et al., 2020).   Several cross-cutting recommendations have also been made including the need for commitment to eliminating disparities at the leadership level, proactive partnerships with disadvantaged communities, and consistent monitoring of progress and provision of feedback (Alcaraz et al., 2020). In addition, the critical need for contextually informed, multi-level, multi-component strategies that target patients, providers, health systems and communities to address health equity in cancer screening and care was renewed during the COVID19 pandemic (Carethers & Doubenl, 2020). Throughout the pandemic, underrepresented minorities, marginalized communities, undocumented individuals, and the under- or uninsured were disproportionately affected by COVID-19 (Siker et al., 2020). The intersections between inequities experienced during the COVID19 pandemic and within current systems of cancer care highlighted the need for urgent action toward achieving cancer health equity (Patel, Lopez et al., 2020). |
| 2021 | Following the NCI’s 2012 and 2016 mandates, a five-year review of how cancer centers have implemented COE resulted in recommendations for increased funding of COE efforts as well as creation of accountability structures and common measures of success around inclusive research and health equity to ensure it is embedded across departments and functions (Doykos et al., 2021). Commonalities between successful COE programs included a community-based foundation that engaged individuals who lived and worked in the target community; a period of program development dedicated to eliciting needs, barriers, cultural concerns, and explanatory models of illness regarding cancer; and incorporation of patient navigators or community-based health workers. |
